# Similar DTI-ALPS metrics in Parkinson’s disease and essential tremor: a cross-sectional comparative analysis

**DOI:** 10.1007/s00234-025-03840-6

**Published:** 2025-11-20

**Authors:** Federico Bruno, Antonio Innocenzi, Pierfrancesco Badini, Marco Cella, Chiara Santobuono, Leonardo Pertici, Alessia Catalucci, Gennaro Saporito, Patrizia Sucapane, Pierpaolo Palumbo, Francesco Arrigoni, Antonio Barile, Ernesto Di Cesare, Francesca Pistoia, Alessandra Splendiani

**Affiliations:** 1 University of L’Aquila, L’Aquila, Italy; 2 San Salvatore Hospital, L’Aquila, Italy

**Keywords:** ALPS-index, Essential tremor, Parkinson’s disease, Neuroradiology, Neurodegenerative disorders, Tremor

## Abstract

**Purpose:**

This study investigated glymphatic function in tremor disorders using the diffusion tensor imaging–derived ALPS (analysis-along-the-perivascular-space) index, a non-invasive surrogate of perivascular fluid movement.

**Materials and Methods:**

Fourty-three patients with tremor-dominant Parkinson’s disease (PD, n=22) or essential tremor (ET, n=21), aged 50–75, were retrospectively compared with 18 age-matched healthy controls (HC) imaged with the same 3 T MRI protocol. Subjects with significant cognitive impairment, white matter disease, other CNS disorders, or poor-quality diffusion data were excluded. ALPS indices were calculated from projection and association fibre regions.

**Results:**

Both PD (mean ALPS = 1.34 ± 0.19) and ET ( 1.31 ± 0.13) groups exhibited 12 significantly lower ALPS values than HC ( 1.54±0.11 P < 0.05), but no difference was 13 found between PD and ET.

In PD, lower ALPS correlated with greater tremor severity and older age, particularly in the right hemisphere (R²=0.80, p=0.003). ET showed weaker clinical associations though MoCA correlated positively with right-hemisphere ALPS (ρ=0.462, p=0.018).

**Conclusion:**

The findings indicate perivascular diffusivity is reduced in both tremor-dominant PD and ET, supporting a role for glymphatic dysfunction in their pathophysiology, though larger, prospective studies are needed to validate this observation.

**Supplementary Information:**

The online version contains supplementary material available at 10.1007/s00234-025-03840-6.

## Introduction

The glymphatic system, so named for its dependence on astroglial Aquaporin-4 (AQP4) water channels and its functional similarity to the peripheral lymphatic system, plays a crucial role in cerebrospinal fluid (CSF) dynamics and brain homeostasis [1]. Through ion exchange and osmotic gradients, these structures mediate fluid movement from the bloodstream into the ventricular system and regulate molecular entry into the CSF [2].

At the cortical surface, cerebral arteries extend into pial arteries that traverse the CSF-filled subarachnoid space and the subpial space. As these arteries penetrate deeper into the brain, they form arterioles that give rise to perivascular (Virchow–Robin) spaces—fluid-filled channels bordered by leptomeningeal cells and astrocytic endfeet rich in AQP4 [3]. This system has been widely described as the brain’s primary waste clearance pathway, facilitating the elimination of macromolecules, cellular debris, and metabolic waste products from the interstitial fluid to the CSF and ultimately into the systemic circulation.

This system is particularly important in several neurological conditions and particularly in the context of neurodegenerative diseases, where the accumulation of misfolded proteins and cellular debris contributes to neuronal dysfunction and cell death.

The concept of the glymphatic system was first supported by Iliff et al. [1]using two-photon fluorescence microscopy with tracers introduced into the subarachnoid space. More recently, glymphatic activity has been visualized using intrathecal administration of gadolinium-based contrast agents and dynamic MRI [4].

Although invasive tracer techniques remain the gold standard for quantifying CSF–interstitial fluid dynamics, their limited feasibility in humans has spurred the development of surrogate magnetic‑resonance‑based biomarkers [5, 6].

To evaluate glymphatic function in humans, magnetic resonance imaging techniques have been developed. While contrast-enhanced MRI can measure CSF influx and efflux rates, it is invasive and has safety concerns.

In 2017, Taoka et al. [4, 7, 8] introduced a non-invasive MRI technique—Diffusion Tensor Imaging Analysis Along the Perivascular Space (DTI–ALPS)—to assess glymphatic function by evaluating anisotropic water diffusivity in specific brain regions. This method compares the mean diffusivity along the *x*-axis in projection and association fiber areas with diffusivity along the y- and z-axes, yielding the ALPS index:

### ALPS index = mean (Dxproj, Dxassoc)/mean (Dyproj, Dzassoc)

A reduced ALPS index is interpreted as impaired glymphatic transport along perivascular spaces.

A growing body of clinical research now links diminished ALPS indices to neurodegenerative proteinopathies. Studies have shown that reduced glymphatic activity is associated with increased amyloid-beta plaque deposition and neuroinflammation, key hallmarks of Alzheimer’s disease [1, 4, 9].

These findings align with experimental data showing that glymphatic dysfunction exacerbates the intracerebral accumulation of amyloid-β, tau, and α-synuclein—hallmark proteins in AD and PD pathogenesis [9].

Beyond classical neurodegeneration, ALPS alterations have been documented in vascular and inflammatory conditions. In subacute ischemic stroke, impaired ALPS indices in corticospinal tracts correlate with Fugl-Meyer motor scores and fractional anisotropy, supporting a link between glymphatic stasis, cytotoxic oedema, and motor dysfunction. Perturbations in perivascular diffusivity have also been reported in idiopathic normal-pressure hydrocephalus, cerebral small-vessel disease, major depressive disorder, and high-grade glioma, highlighting the method’s translational reach [10–15].

PD is a chronic progressive course disease clinically defined by tremor, rigidity, bradykinesia and postural instability. Neuropathologically, it is characterized by severe degenerative changes in the substantia nigra (pars compacta) and pigmented nuclei of the brainstem. Characteristic cellular inclusion due to misfolded α-synuclen, called Lewy Bodies (LB), can be found in the residual neurons [16, 17].

In recent years, the study of the glymphatic system in such pathophysiological conditions has gained ground, since the glymphatic system itself is considered to be primarily responsible for the clearence of metabolic waste products. Hence, impairment of glymphatic system may lead to reduced clearance of α-syn and its consequent accumulation, which plays a critical role in the pathogenesis of PD [18]. Even more recently, several studies have attempted to apply non-invasive assessment by MRI studies based on DTI sequences and using the ALPS index and they found a reduction of this index in patients with PD. Clinically, ALPS index was found to correlate with PD disease severity, motor symptoms, and autonomic function, and can predict the deterioration of motor skills and cognitive function [5, 6, 19].

ET, often mislabelled as “benign”, “familial”, “senile”, is the most common movement disorder in the adult population. In the second half of the 20th century, the term ET appeared in numerous medical publications indicating a form of kinetic tremor, usually familial, with an unknown cause. This conception of the condition remained intact for decades but has recently been called into question. The etiology of ET has yet to fully elucidated, largely due to the complexity and heterogeneity of its underlying pathological mechanism [20, 21]. Indeed, a growing body of neuropathological and advanced imaging studies now supports its classification along the neurodegenerative spectrum, with cerebellar Purkinje cell loss, abnormal protein aggregation, and aberrant oscillatory activity within the dentato‑rubral network. Although ET has historically been viewed as a benign monosymptomatic tremor, converging neuroimaging and neuropathological data point toward degenerative changes within the cerebellar–thalamic circuitry and, including Lewy-related pathology [20–24].

In this context, the present study aims to evaluate and compare the ALPS index among patients with ET, PD, and healthy controls, to explore potential glymphatic dysfunction and its clinical relevance.

## Materials and methods

We undertook a review of our prospectively maintained MRgFUS database and hospital electronic health records to identify patient who underwent unilateral ventralis intermedius nucleus (VIM) thalamotomy with MR‑guided focused ultrasound (MRgFUS) for tremor control between April 2018 and July 2023 in a single institution.

We included in the analysis all patients aged 50–75 years who were treated for right-hand tremor. Demographic information including sex, age, underlying pathology, and disease duration were recorded.

Patients were excluded if they met any of the following: incomplete clinical records and missing data, presence of other neurodegenerative disease; prior or concomitant central‑nervous‑system pathology (e.g., multiple sclerosis, psychiatric disorders, large malacic cavities, space‑occupying lesions, vascular malformations, or prior intracranial haemorrhage); Montreal Cognitive Assessment (MoCA) < 10; extensive white‑matter hyperintensities (Fazekas ≥ 2); poor‑quality diffusion‑tensor images (e.g., motion, susceptibility, low resolution); or left‑handedness.

Fourty-three patients satisfied all criteria (22 PD, 21 ET). A control cohort comprised 18 neurologically healthy volunteers (11 female; age 30–50 years) who had been prospectively enrolled in a prior study by our group investigating the ALPS index with the same 3 T scanner and identical DTI protocol [13]. The controls were not age‑ or sex‑matched to the patient cohort, serving solely as a reference for diffusivity metrics.

### Clinical evaluation

According to our Institution protocol, all patients screened for protocol are submitted to a clinical assessment by a team of neurologists specialized in movement disorders [25–27]. The diagnostic criteria for ET included criteria reported in literature including clinical features of bilateral action tremor of the hands and forearms and the absence of other neurologic signs and exclusion of alternative diagnosis. Other criteria for essential tremor included long disease duration (more than 3 years), a positive family history of essential tremor, and beneficial response to alcohol [28].

The evaluation relies on a detailed neurologic examination to identify specific features of the tremor, including its frequency, amplitude, pattern, and distribution, and to identify other neurologic findings if present. Precipitating, aggravating, or relieving factors such as caffeine, alcohol, medications, exercise, fatigue, or stress should be elicited; a.

The cornerstone of the neurological examination is the quantification of tremor severity and global cognition. Tremor is rated with the Fahn–Tolosa–Marín Clinical Rating Scale for Tremor (CRST), which captures rest, postural, and kinetic components as well as tremor‑related disability. Cognitive status is screened with the 30‑point Montreal Cognitive Assessment (MoCA), a sensitive tool for detecting mild cognitive dysfunction in PD and ET. Part A of the CRST—focusing on tremor amplitude of the upper limbs—was prospectively designated as the primary outcome measure for both pre‑ and post‑procedural evaluations. This strategy not only standardizes assessment across PD and ET but also concentrates on the motor domain most relevant to the patient’s functional gains.

After baseline assessment and MRgFUS treatment, patients are scheduled for systematic follow‑up visits at 1, 6, and 12 months, and annually thereafter. Each visit replicates the pre‑treatment evaluation, including MoCA, CRST, and brain MRI with the same protocol, permitting longitudinal comparison.

### MRI data acquisition

All MRI scans were performed on a 3.0 Tesla scanner (Discovery 750; GE Healthcare, Milwaukee, WI, USA) using a 32-channel head coil. The imaging protocol included, along with standard morphological sequences, Diffusion‑tensor imaging (DTI): spin‑echo echo‑planar imaging (Acquisition parameters: repetition time (TR) = 8,000 ms; echo time (TE) = minimum; flip angle = 90; field of view = 256 × 256 mm²; matrix = 128 × 128; slice thickness = 3 mm (no gap); b = 1,000 s/mm²; 30 non‑collinear diffusion directions plus one b0 volume) and Susceptibility Weighted Imaging (SWI) (acquisition parameters: TR = 33.7 ms; first TE = 4.556 ms; echo spacing = 3.648 ms; final TE = 30.092 ms; flip angle = 20; field of view = 240 × 240 mm²; matrix = 416 × 384; slice thickness = 2 mm).

Clinically, DTI is acquired primarily to refine VIM targeting during surgical planning, whereas the susceptibility-weighted sequence is employed post-operatively to visualise and delineate the thalamotomy lesion.

## Image processing and analysis

DTI data were processed with DSI Studio (build 2023‑Jan‑15). After conversion to NIfTI and SRC formats, volumes were corrected for eddy‑current–induced distortions and head motion, skull‑stripped, and reconstructed with generalized q‑sampling imaging to generate individual fib files.

Assessment of glymphatic function followed Taoka et al.’s “analysis along the perivascular space” (ALPS) method [4, 7, 8]. A single axial slice at the body of the lateral ventricles—where perivascular spaces run predominantly in the right‑to‑left (x) direction—was selected. In both hemispheres, two spherical regions of interest (ROI; 5 mm diameter) were manually placed on color‑coded fractional‑anisotropy maps by a neuroradiologist (>10 years’ experience) blinded to all clinical data:

- Projection‑fibre ROI in the corona radiata (blue‑coded fibres, z‑axis orientation).
- Association‑fibre ROI in the superior longitudinal fasciculus (green‑coded fibres, y‑axis orientation).

For each ROI, diffusivities along the three orthogonal axes were extracted, yielding six scalar values:

- Projection fibres– Dxx‑proj, Dyy‑proj, Dzz‑proj.
- Association fibres– Dxx‑assoc, Dyy‑assoc, Dzz‑assoc.

The ALPS index was calculated as: ALPS index = mean (Dxxproj, Dxxassoc)/mean (Dyyproj, Dzzassoc), and the mean value of the two sides was considered.

### Statistical analysis

Statistical analyses were performed with MedCalc (MedCalc Software Ltd., Ostend, Belgium; v.20.2). Continuous variables were assessed for normality using the Kolmogorov–Smirnov test. Data are expressed as mean ± standard deviation (SD) when normally distributed and as median and interquartile range when skewed.

Differences in ALPS indices between healthy controls (HC), essential tremor (ET), and Parkinson’s disease (PD) groups were evaluated using one-way analysis of variance (ANOVA), followed by Bonferroni-adjusted post-hoc tests to account for multiple comparisons. Relationships between the ALPS index and clinical variables (disease duration, CRST Part A and total, MoCA) were examined with Pearson correlation coefficients for normally distributed pairs and Spearman’s ρ when assumptions of normality were violated. Statistical significance was set at a two‑tailed *p* < 0.05. Multiple linear regression analyses were performed to evaluate the relationship between ALPS indices (mean, left, and right hemispheres) and clinical variables, including disease duration, age, tremor severity (FTM scores), and cognitive status (MoCA). Separate models were constructed for the Parkinson’s disease (PD) and essential tremor (ET) cohorts.

## Results

### Demographic and clinical characteristics

Baseline demographic and clinical characteristics are summarized in Table 1.

**Table 1:** Baseline demographic and clinical characteristics of study cohort

Group	*n*	Age, y (mean ± SD)	Sex (M/F)	SIDE TREATED (*R*/L)	Disease duration, y (median ± 95% CI)	MoCA (mean ± SD)
ET	21	65.3 ± 7.83	9/12	6/15	20 (15.3–40.5)	23.4 ± 2.8 (95% CI 21.6–25.2)
PD	22	64.2 ± 8.51	13/9	8/14	6.5 (5–9)	24.7 ± 3.2 (95% CI 22.9–26.4)
HC	18	53.0 ± 4.21	7/11		—	

No statistically significant differences were detected between the PD and ET cohorts with respect to age (t-test, *p* = 0.19) or sex distribution (χ², *p* = 0.36). Disease duration, however, was significantly longer in the ET group (median 20 years, 95% CI 15.3–40.5) than in the PD group (median 6.5 years, 95% CI 5–9, *p* < 0.001). Tremor severity, quantified with the CRST score, was likewise higher in the ET cohort (41.1 ± 14.7, range 17–44) than in the PD cohort (23.9 ± 14.8, range 11–63; *p* = 0.004). Considering the Part A of the treated limb, baseline CRST values were comparable (mean values ET 5.8 ± 2.2, PD 5.6 ± 1.9, *p* = 0.53). Global cognitive performance, as assessed by the MoCA, did not differ between groups (*p* = 0.68).

## ALPS index across study groups

### ALPS indices measured in the right and left hemispheres are reported in Table 2

**Fig. 1:**
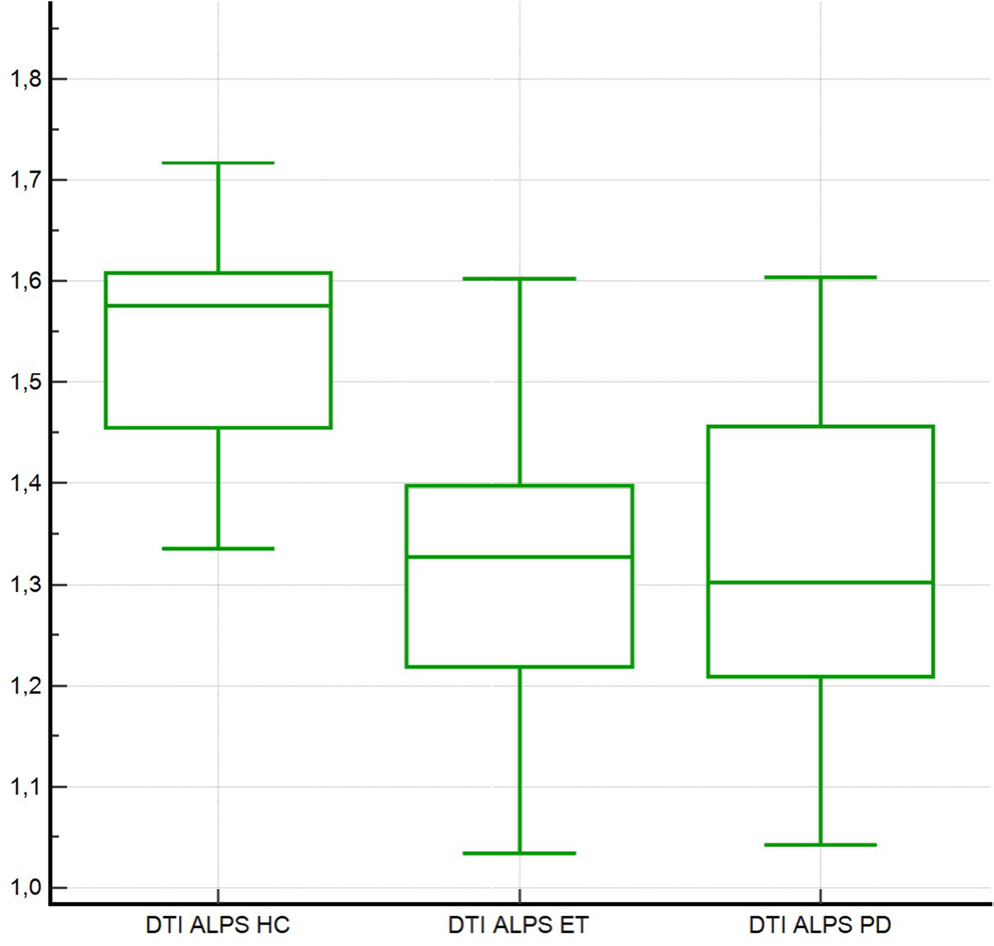
Comparison of DTI-ALPS indices in healthy controls (HC), essential tremor (ET), and Parkinson’s disease (PD). Box-HC exhibited higher ALPS indices compared with both ET and PD showing overlapping distributions, with lower median values than HC

Mean ± SD ALPS indices for the three cohorts were PD = 1.34 ± 0.19 (95% CI 1.25–1.42), ET = 1.31 ± 0.13 (95% CI 1.25–1.37), and HC = 1.54 ± 0.11 (95% CI 1.48–1.60). Post-hoc comparisons with Bonferroni adjustment demonstrated that both patient groups exhibited lower ALPS values than HC (each *P* < 0.05), whereas the ET–PD comparison did not reach statistical significance (*P* = 0.67) (Fig. 1).

In the ET cohort, regression analyses of the ALPS indices (mean, left, and right hemispheres) did not yield any statistically significant associations with clinical predictors. The overall explanatory power of the models was low (R² ranging from 0.37 to 0.42; all models nonsignificant). Among the predictors, age, disease duration, and tremor severity consistently showed negative regression coefficients in some models, but none reached statistical significance. The most notable trend was observed for tremor severity in the left hemisphere (β = − 0.069, *p* = 0.163).

In correlation analysis age, tremor intensity, and disease duration did not show significant negative correlations with ALPS, while cognitive performance (MoCA) correlated positively with the right ALPS index (ρ = 0.462, *p* = 0.018).

In PD patients, for the mean ALPS index, the regression model was statistically significant and explained approximately 74% of the variance (R² = 0.74, adj. R² = 0.61). Among the predictors, tremor severity (CRST score) showed a significant negative association with ALPS values (β = − 0.044, *p* = 0.048).

For the right hemisphere ALPS index (ALPS_R_PD), the regression model was even stronger (F [5, 10] = 8.21, *p* = 0.003), accounting for 80% of the variance (R² = 0.80, adj. R² = 0.71). In this model, both tremor severity (β = − 0.055, *p* = 0.029) and age (β = − 0.0087, *p* = 0.044) emerged as significant negative predictors. By contrast, the left hemisphere ALPS index (ALPS_L_PD) showed a poor model fit (R² = 0.41, adj. R² = 0.12; F [5, 10] = 1.42, *p* = 0.299), with no significant predictors.

Correlation analyses were consistent with these findings. Significant negative correlations were observed between age and both the mean ALPS index (ρ = − 0.339, *p* = 0.025) and the right hemisphere ALPS index (ρ = − 0.570, *p* = 0.004). Similarly, tremor severity demonstrated negative correlations with ALPS indices, reaching significance for the right hemisphere (ρ = − 0.513, *p* = 0.020).

**Table 2:** Mean ALPS-index values (± SD) for the right and left hemispheres across the three groups—Essential Tremor (ET), Parkinson’s Disease (PD), and Healthy Controls (HC)

	RIGHT	LEFT	*p*-value
ET	1.31 ± 0.14 (95% CI 1.25 − 1.37)	1.33 ± 0.17 (95% CI 1.26 − 1.41)	0.52
PD	1.34 ± 0.20 (95% CI 1.25 − 1.42)	1.39 ± 0.16 (95% CI 1.32 − 1.47)	0.09
HC	1.54 ± 0.12 (95% CI 1.48 − 1.60)	1.50 ± 0.12 (95% CI 1.44 − 1.56)	0.22

## Discussion

Pathologically, Parkinson’s Disease (PD) is characterized by the degeneration of dopaminergic neurons in the substantia nigra and the abnormal aggregation of α-synuclein, forming Lewy bodies. The glymphatic system is thought to facilitate clearance of α-synuclein, implicating its dysfunction in the progression of PD [9, 17, 19, 29, 30]. Conversely, the pathological basis of Essential Tremor (ET) is less well understood. While traditionally considered a benign, functional disorder, increasing evidence supports a neurodegenerative component in ET [21, 22].

Histological studies have demonstrated Purkinje cell loss and structural cerebellar changes in ET, which—along with cognitive, psychiatric, sensory, and sleep disturbances—support its classification among neurodegenerative conditions [22, 24]. Epidemiologically, late-onset ET is associated with an increased risk of developing both Alzheimer’s disease and Parkinson’s disease. Furthermore, iron accumulation, a hallmark of several neurodegenerative diseases, has also been observed in ET [20, 21, 24, 31].

Despite these clues, direct evidence linking ET to glymphatic dysfunction is lacking. To our knowledge, this is among the few studies to explore ALPS index values in both ET and PD populations.

In this single‑center exploratory study, we provide convergent evidence that the perivascular diffusivity captured by the ALPS index is attenuated in two clinically distinct tremor syndromes—tremor‑dominant Parkinson’s disease and essential tremor—when compared with neurologically healthy individuals.

Several recent studies have examined glymphatic metrics in movement disorders. Gu et al. analyzed 124 PD, 74 ET, and 106 healthy controls and found a markedly lower ALPS index and a higher MRI‑visible perivascular‑space (EPVS) burden in PD relative to both ET and controls; ALPS values inversely correlated with UPDRS‑III scores in PD, but no ALPS differences emerged between ET and controls [6].

McKnight et al. reported a reduced ALPS index in PD versus ET among 181 DBS candidates, with additional associations between lower ALPS, advancing age, and greater white‑matter disease [5].

In a mixed cross‑sectional and longitudinal analysis of the PPMI dataset, He et al. demonstrated negative correlations between ALPS and age, disease duration, Hoehn–Yahr stage, and MDS‑UPDRS‑III [19].

Collectively, these investigations converge on the notion of glymphatic compromise in PD, whereas findings in ET remain inconclusive—positioning the present study, which shows ALPS reduction in both disorders, as an important extension of the literature.

Consistent with accumulating evidence in Parkinson’s disease, we observed significantly lower ALPS values in PD compared with healthy controls. Crucially, ET patients exhibited a comparable reduction, contradicting earlier work that reported preserved or only mildly diminished ALPS indices in ET.

Methodological and clinical distinctions likely account for the discrepancy. Our patients were older (≈ 68 years), manifested longer disease duration, and were selected for MRgFUS specifically because of medically refractory, severe tremor. By contrast, prior series enrolled broader outpatient samples.

Interestingly, in McKnight et al. [5], patients underwent MRI under sedation prior to deep brain stimulation, which may have influenced ALPS values, as glymphatic activity increases during sleep [32, 33]. In our study, all MRIs were performed in awake patients, which could reduce ALPS sensitivity but better reflect daytime baseline glymphatic function.

We also controlled for white matter hyperintensities (WMH) by excluding individuals with Fazekas scores ≥ 2. This is important, as WMH burden inversely correlates with ALPS index, and failure to account for this may confound group comparisons [11, 29, 34].

In contrast to several prior studies that demonstrated significant associations between reduced DTI‑ALPS index and clinical or cognitive severity in PD patients, our cohort did not exhibit similar correlations with age, disease duration, or CRST scores.

Regarding ET patients, our findings are partially in line with a single recent paper published in literature by Qin et al., who reported no significant correlations between DTI-ALPS index and clinical variables in pure ET, while reduced ALPS index was evident only in ET-plus and correlated inversely with disease duration in this subgroup [35].

The Parkinson’s disease subgroup in our cohort is likewise atypical, comprising exclusively tremor‑dominant (“tremorgenic”) PD selected for lesioning rather than conventional deep‑brain stimulation or medical optimisation. Extrapolation to akinetic‑rigid or mixed PD phenotypes, community‑based populations, milder tremor, or non‑dominant‑hand targets is therefore uncertain. Moreover, healthy controls were younger and predominantly female; age‑related diffusivity changes may partly account for case–control differences despite prior reports of minimal age influence on ALPS.

ALPS quantification remains sensitive to ROI placement, diffusion‑tensor reconstruction strategy, software (e.g. DSI Studio vs. FSL or MRIstudio), field‑strength, and head motion. To mitigate confounds, we excluded scans with pronounced artefact and individuals with moderate‑to‑severe white‑matter hyperintensity burden (Fazekas ≥ 2), given the established negative correlation between WMH volume and ALPS. Nevertheless, operator‑defined ROIs introduce residual subjectivity despite good intra‑ and inter‑observer repeatability reported elsewhere [36].

While we used manually placed, spherical ROIs—as per the original description—other investigators have adopted fully automated, atlas‑based positioning that anchors ROIs to predefined white‑matter parcellations [8]. Such methodological variability can generate systematic offsets and inflate inter‑study dispersion, thereby hampering reproducibility and meta‑analytic synthesis. Harmonisation of ROI strategy, ideally through consensus atlases and open‑source pipelines, is needed to permit valid cross‑centre comparison. Automatic or atlas‑based ROI placement could enhance consistency, but requires rigorous validation against expert‑guided methods [37].

Laterality of measurement is also a crucial consideration when assessing DTI ALPS. Studies in healthy adults demonstrated that the left ALPS index is influenced by handedness and, in females, by language lateralization, independent of disease. Such functional lateralization must be considered when interpreting ALPS asymmetry, as it can reflect normative brain traits rather than pathology [38].

A metanalysis conducted by Costa et al. across multiple DTI‑ALPS studies in PD and Alzheimer’s disease highlighteda a marked lateralization (hemispheric asymmetry) in PD, a feature not present in Alzheimer’s [39]. Recent results by Li et al. demonstrated distinct hemispheric patterns in Parkinson’s patients with left- or right-onset disease, including altered asymmetry indices that correlate with motor severity [40, 41]. This aligns with our cohort’s directional trend of reduced ALPS in the hemisphere contralateral to the more affected side—albeit without statistical significance. Moreover, our results showed that negative correlations with tremor severity and age were strongest in the right hemisphere, while the left hemisphere model did not yield significant associations. This pattern reinforces the notion that ALPS lateralization in PD is not merely a reflection of normative brain traits but a disease-linked phenomenon, closely tied to motor dysfunction.

Beyond these implementation details, a more fundamental critique concerns construct validity: the ALPS index derives from anisotropic water diffusion rather than direct measurement of bulk CSF flow, and therefore may be influenced by axonal architecture, extracellular oedema, microvascular permeability, or partial‑volume effects. A high ALPS index merely reflects predominant radial diffusion at the level of the lateral ventricle body. It should not be directly equated with glymphatic efficiency. As Taoka et al. recently noted, ALPS should be interpreted as “increased” or “decreased,” and not conclusively labeled as glymphatic function or dysfunction. Furthermore, the ALPS index alone is insufficient to capture the complexity of interstitial fluid dynamics. Whether the metric truly captures glymphatic throughput—or instead reflects a composite of tissue properties adjacent to perivascular spaces—remains a matter of debate and represents a key area for methodological refinement and physiological validation. A multimodal approach, combining ALPS with advanced MRI techniques (e.g., intrathecal contrast studies or perfusion imaging), is likely necessary to provide a comprehensive assessment of glymphatic function [7, 39, 42].

There are indeed also limitations of the present study that deserve mention.

Although our cohort represents the largest series of MRgFUS‑screened tremor patients investigated with ALPS to date, the absolute numbers—particularly when stratified by diagnosis—remain modest. The study should therefore be regarded as hypothesis‑generating rather than definitive; small‑sample inference inflates type‑I and type‑II error risk and precludes robust subgroup analyses (e.g., sex‑specific or disease‑duration effects).

The retrospective, single‑centre design is inherently susceptible to selection bias and missing data. Only patients with complete imaging and clinical documentation were included, potentially enriching the sample for individuals with favourable follow‑up adherence or superior image quality. Prospective enrollment would mitigate these biases.

Finally, vascular risk factors, sleep quality, and medication effects (e.g., dopaminergic agents, beta‑blockers) were not systematically recorded, yet each may modulate glymphatic flow.

## Conclusions

In this study, we confirmed previous literature observations that the ALPS index is reduced in patients with Parkinson’s Disease (PD) compared to healthy controls, in line with previous findings. Notably, we also observed that patients with Essential Tremor (ET) exhibit similarly reduced ALPS index values, suggesting that the glymphatic system may be involved in ET as well, particularly in patients with more severe, treatment-resistant tremor.

These findings support the notion that ET may not be solely a functional or electrical disorder, but rather a condition with potential structural and degenerative substrates. However, due to methodological limitations and variability in ALPS assessment, further studies with larger, prospective cohorts and multimodal imaging protocols are needed to validate these observations.

Ultimately, while the ALPS index offers a promising non-invasive tool for evaluating glymphatic activity, its interpretation should remain cautious, and it should ideally be used in conjunction with complementary imaging and clinical data to better understand interstitial fluid dynamics and neurodegenerative processes.

## Supplementary Information

Below is the link to the electronic supplementary material.

Supplementary Material 1 (JPG 319 KB)

## Data Availability

No datasets were generated or analysed during the current study.
