# Supplementary figures and images for: Similar DTI-ALPS metrics in Parkinson’s disease and essential tremor: a cross-sectional comparative analysis
Source: Neuroradiology. 2025 Nov 20;68(2):483–91. doi: 10.1007/s00234-025-03840-6 (PMC13021799; doi:10.1007/s00234-025-03840-6)

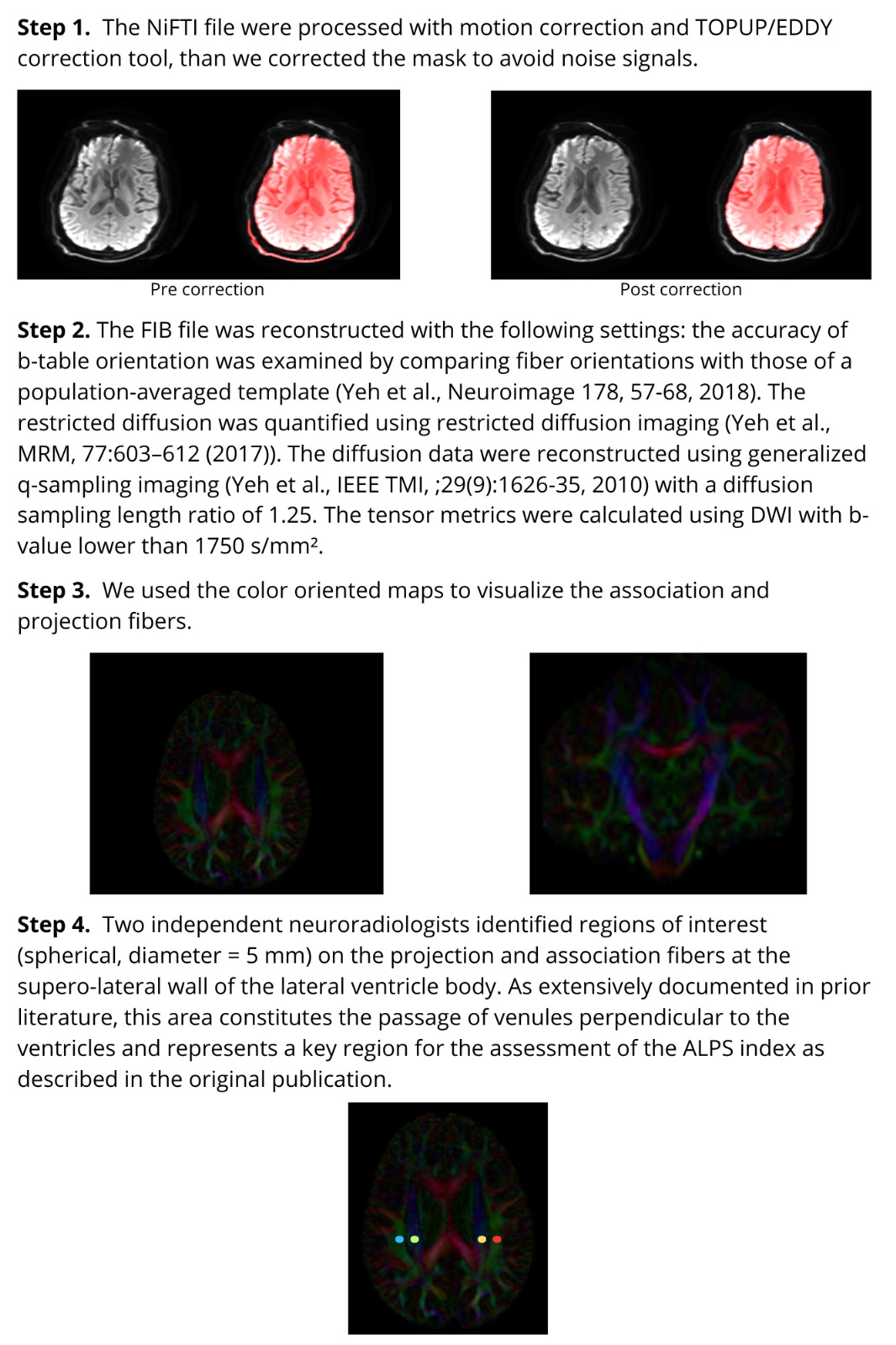

Supplement: Supplementary file 1 — Supplementary Material 1 (JPG 319 KB) [file 234_2025_3840_MOESM1_ESM.jpg]
